# RIOK3 and Its Alternatively Spliced Isoform Have Disparate Roles in the Innate Immune Response to Rift Valley Fever Virus (MP12) Infection

**DOI:** 10.3390/v14092064

**Published:** 2022-09-17

**Authors:** Thomas C. Bisom, Luke A. White, Jean-Marc Lanchy, J. Stephen Lodmell

**Affiliations:** 1 Department of Chemistry and Biochemistry, University of Montana, Missoula, MT 59801, USA; 2 Division of Biological Sciences, University of Montana, Missoula, MT 59801, USA; 3 Center for Biomolecular Structure and Dynamics, University of Montana, Missoula, MT 59801, USA

**Keywords:** Rift Valley fever virus, RIOK3, alternative splicing, NFκB, innate immunity

## Abstract

Rift Valley fever virus (RVFV) is a pathogenic human and livestock RNA virus that poses a significant threat to public health and biosecurity. During RVFV infection, the atypical kinase RIOK3 plays important roles in the innate immune response. Although its exact functions in innate immunity are not completely understood, RIOK3 has been shown to be necessary for mounting an antiviral interferon (IFN) response to RVFV in epithelial cells. Furthermore, after immune stimulation, the splicing pattern for RIOK3 mRNA changes markedly, and RIOK3′s dominant alternatively spliced isoform, RIOK3 X2, exhibits an opposite effect on the IFN response by dampening it. Here, we further investigate the roles of RIOK3 and its spliced isoform in other innate immune responses to RVFV, namely the NFκB-mediated inflammatory response. We find that while RIOK3 is important for negatively regulating this inflammatory pathway, its alternatively spliced isoform, RIOK3 X2, stimulates it. Overall, these data demonstrate that both RIOK3 and its X2 isoform have unique roles in separate innate immune pathways that respond to RVFV infection.

## 1. Introduction

**Figure 1:**
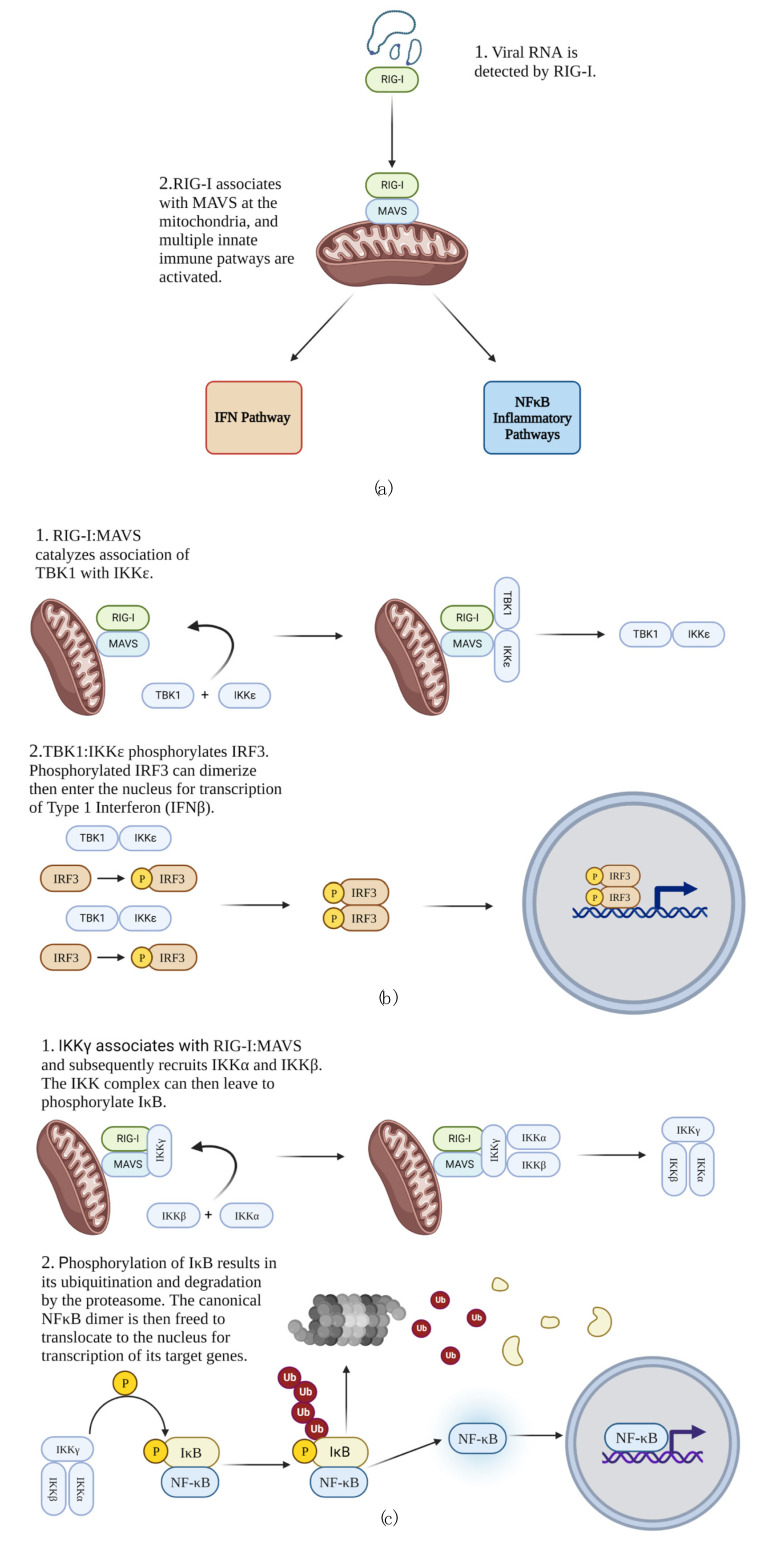
RIG-I-mediated innate immune response to RNA virus infection. (**a**) Innate immune pathways activated in response to RNA viral genome. (**b**) IFN response. (**c**) Canonical NFκB pathway.

When RNA viruses are detected by the cell, a series of intricate innate immune responses is initiated to clear the virus, warn neighboring cells of viral presence, and recruit more specialized immune cells to the site of infection. Retinoic acid-inducible gene I (RIG-I), melanoma differentiation factor 5 (MDA5; also known as IFIH1), and laboratory of genetics and physiology 2 (LGP2) are intracellular receptors capable of detecting RNA viruses in the cytosol, and of these, RIG-I is currently the most well-understood (reviewed in [1]). Upon binding viral RNA in the cytoplasm, RIG-I’s caspase activation and recruitment domain (CARD) is released from an inhibitory association with its helicase domain [2,3]. Concurrently, immune activated E3 ubiquitin ligases impart K63-linked polyubiquitin chains to several key mediators of the immune response, including RIG-I [4,5,6,7,8]. RIG-I is then recruited to the mitochondria [9,10] where it associates with the mitochondrial antiviral signaling protein (MAVS; also known as IPS-1, VISA, and Cardif) via CARD domains of both RIG-I and MAVS [11]. This activated complex of RIG-I and MAVS (RIG-I:MAVS) serves as the intersection of multiple innate immune pathways that are simultaneously activated to respond to the infection, notably the interferon (IFN) and nuclear factor κB (NFκB) pathways (Figure 1a) [12,13].

Although these pathways are ideally designed to elicit a response for clearing the virus and recruiting other immune cells to fight pathogens, RNA viruses have evolved strategies to evade, exploit, or dysregulate these pathways. This has been highlighted recently with SARS CoV-2 infections that caused the 2019 COVID pandemic, where in many severe disease cases, a decreased IFN response but exaggerated inflammatory response was observed [14,15]. Therefore, understanding innate immune responses to viruses and how they are dysregulated or co-opted during viral infection can help with countermeasures to current and future pandemics and epidemics.

Organisms and biological agents that pose the highest risk to national security and public health are designated by the National Institute of Allergy and Infectious Disease (NIAID) as category A pathogens. Rift Valley fever virus (RVFV; order Bunyavirales, family Phenuiviridae, genus Phlebovirus [16]) is a tripartite ambisense RNA virus [17,18,19] designated by the NIAID as a category A pathogen because of its potential for devastating outbreaks among humans and livestock [20]. Because of its potential to cause harm to humans and livestock, RVFV is also considered a CDC/USDA Overlap Select Agent requiring stringent containment and regulatory measures. However, the attenuated RVFV strain MP12 can be handled safely in BSL2 containment and is exempt from the Select Agent list. MP12 is derived from the wt pathogenic isolate ZH548, but it harbors mutations in all three genomic segments that mitigate its pathogenicity [21]. While differences in MP12 vs. pathogenic isolates warrant additional corroboration of experimental results obtained with MP12, the basic virological attributes of MP12, including its cytopathogenicity in cell culture, make it a logistically tractable and cost-effective surrogate for initial studies of RVFV and its interactions with human cells. It is therefore the strain of choice for routine laboratory experiments involving viral infection in many laboratories studying RVFV, including ours.

Intracellularly, RVFV RNA is detected primarily by the receptor RIG-I [22,23]. We have previously shown that the atypical kinase RIOK3 plays an important role in mediating the RIG-I-based interferon pathway response to RVFV in epithelial cells. With CRISPR/Cas-mediated knockout (KO) or siRNA knockdown of RIOK3, RVFV titer increased significantly, suggesting RIOK3 has an antiviral role in the cell in response to RVFV infection in cell culture [24]. Moreover, we observed that during RVFV infection, RIOK3 mRNA is alternatively spliced, primarily to its X2 isoform that encodes a truncated protein, which was seen as a potential strategy employed by RVFV to subvert the antiviral response [24,25,26].

To date, RIOK3 has been shown to be involved in both the IFN and NFκB pathways. During activation of the IFN pathway, RIG-I:MAVS promotes formation of a complex of two kinases: TANK-binding kinase 1 (TBK1) and inhibitor of nuclear factor κB kinase subunit ε (IKKε). The TBK1:IKKε kinase complex phosphorylates monomeric interferon regulatory factor 3 (IRF3) in the cytosol [27,28,29]. Phosphorylated IRF3 dimerizes, then enters the nucleus to serve as a transcription factor for IFNβ and other relevant genes (Figure 1b) [30,31]. A primary purpose of transcribing IFNβ is to activate the Janus kinase/signal transducer and activator of transcription (JAK/STAT) pathway in autocrine and paracrine manners, which results in transcription of interferon stimulated genes (ISGs) that promote the cellular antiviral state [32,33,34,35,36,37,38]. RIOK3 has been shown to act as an adapter linking TBK1 to IRF3 and therefore likely aids in mounting the IFN response [39]. However, RIOK3 has also been shown to have roles in negative regulation of the IFN pathway in different experimental contexts [40,41].

Simultaneous and in parallel to activation of the IFN pathway, NFκB pathways are activated to trigger an inflammatory response that recruits more specialized immune cells to the area of infection and also establishes an environment unfavorable for the virus [42]. NFκB proteins are a family of transcription factors comprising p50, p65 (also called RELA), c-REL, p52, and RELB. Of these, p50, p65, and c-REL, exist as dimers (p50:p65, p50:c-REL, and p50:p50) in the cytosol that are kept transcriptionally inactive by their associations with members of the inhibitor of κB (IκB) family [43,44,45,46,47]. During RIG-I-mediated activation of the canonical NFκB pathway, the adapter protein IKKγ (also known as NEMO) is recruited to RIG-I:MAVS, and the kinases IKKα and IKKβ then form a complex with IKKγ and are activated by phosphorylation [12,48,49,50,51,52]. The activated IKK complex then phosphorylates IκB bound to canonical NFκB dimers, which leads to K48-linked polyubiquitination and subsequent degradation of IκB, enabling the NFκB dimers to enter the nucleus to promote transcription of target genes (Figure 1c) [51,53,54,55,56,57]. One consequence of this pathway is transcription and translation of TNFα. TNFα protein can then be released from the cell and specifically activates the canonical NFκB pathway in neighboring cells in a paracrine fashion. Although in this context the NFκB pathway is not activated through RIG-I:MAVS, the mechanism is otherwise similar [58,59,60,61]. Prior experiments testing the role of RIOK3 in the NFκB pathway have primarily investigated its role in the cellular response to TNFα. These studies suggested that RIOK3 negatively regulated the canonical NFκB pathway in response to TNFα protein [62,63]. 

Although there is general consensus that RIOK3 is involved in regulating the antiviral response to RNA viruses, its exact functions are still not clear and vary considerably with different experimental conditions, viruses, and cell types. For example, Shen et al. [41] and Takashima et al. [40] found RIOK3 can negatively regulate the IFN response, but results by Feng et al. [39] and our laboratory [24] suggest it is required for mounting this response. Recently, we showed through RT-qPCR that RIOK3 knockout (KO) cells treated with reagents known to activate the IFN pathway (poly(I:C) and 3p-hpRNA) exhibit decreased expression of IFNβ, suggesting RIOK3 is involved in activating this response [24]. Still, the lack of agreement in the literature concerning RIOK3′s role in the IFN pathway compels more in-depth experimentation to elucidate the functions of this kinase. RIOK3 may in fact have roles in both positive and negative regulation of the IFN response. For example, Willemsen et al. [64] demonstrated that RIOK3 activates RIG-I signaling in response to RVFV but negatively regulated it in response to influenza A infection. Moreover, RIOK3′s apparent functional duality does not appear to only apply to the IFN response. Results by Fenner et al. [63] demonstrated both positive and negative regulatory effects of RIOK3 on the canonical NFκB response. The reported roles of RIOK3 in innate immunity has therefore been context-specific and more studies are indicated to uncover mechanisms of action. 

Here, we discriminate the specific effects of RIOK3 in both the IFN and inflammatory pathways during RVFV MP12 infection. We demonstrate that RIOK3 is required for mounting an IFN response but negatively regulates the NFκB-mediated inflammatory response, and intriguingly, alternative splicing of RIOK3 reverses these effects.

## 2. Materials and Methods

### 2.1. Viruses, Infections and Cell Culture

The attenuated BSL2 laboratory RVFV strain MP12 (kindly provided by Brian Gowen Utah State University, Logan, UT, USA) was used for all infections. Viral stocks were made from Vero cells. After 1 h incubation with virus in Dulbecco’s Modified Eagle Medium (DMEM) containing no serum or antibiotics, media was exchanged with low serum media containing antibiotics (DMEM with 2% fetal bovine serum (FBS) and penicillin + streptomycin (pen/strep)), and the cells were incubated at 37 °C, 5% CO_2_ and harvested 24 h post-infection (h.p.i.). Quantification of infectious dose was performed in TCID50 assays described by Smith et al. [65] with Vero 76 cells, and TCID50/mL values were obtained using the Reed and Muench Method. Human embryonic kidney 293 (HEK 293) cells were used in all other experimental assays, and all cells were grown in DMEM containing 10% FBS and pen/strep. Experiments were initiated when cells were near confluency. RIOK3 KO HEK 293 cells were generated by CRISPR/Cas-mediated genome editing, as described previously [24]. HEK 293 and Vero 76 cells were obtained from ATCC (Manassas, VA, USA). Manipulations of the viruses used in this study are compliant with both the Institutional Biosafety Committee at the University of Montana, Missoula, and NIH requirements in regard to their handling under BSL2 containment conditions. 

### 2.2. Reagents

TNFα was purchased from BioLegend (San Diego, CA, USA). Prior to treatment with TNFα in experimental assays, growth media was removed; cells were washed with PBS, and PBS was exchanged with fresh DMEM media containing 10 or 2% FBS and pen/strep. TNFα was then added such that its final concentration in each well was 20–80 ng/mL, and cell were then incubated at 37 °C, 5% CO_2_ for 4 or 24 h.; see text for specific concentrations and incubation times used in each assay. 

### 2.3. Plasmids, Oligos and Transfections

RIOK3 X2 was expressed in a phRL-CMV backbone as described previously [24]. The pNFκB-Luc *cis* luciferase reporter plasmid was kindly provided by Joel Graff (Montana Technological University, Butte, MT, USA) and is from the Path Detect Series (Agilent, Santa Clara, CA, USA). Each construct was transfected with 1.6 μg DNA per well in 12-well plates. Plasmid transfections were accomplished with Lipofectamine 2000 (Thermo Fisher Scientific, Waltham, MA, USA). In these transfections, growth media was removed, cells were washed with PBS, and PBS in each well was exchanged with a volume of OptiMEM transfection media equal to half the volume of growth media. The cells were then incubated at 37 °C, 5% CO_2_. While the cells were incubating, transfection master mixes were prepared. When preparing Lipofectamine master mixes, Lipofectamine was added to OptiMEM media and incubated at room temperature for 5 to 20 min. Lipofectamine was added to each master mix such that the ratio of the volume of Lipofectamine 2000 to mass of construct transfected would be ~2.5 per well. After incubation of Lipofectamine master mixes, these mixtures were added to DNA construct master mixes comprising construct DNA and OptiMEM, and the resulting transfection mixture was left to incubate at room temperature for 20 min. to allow assembly of DNA: liposome complexes. Transfection mixtures were then dispensed into wells with cells that incubated in OptiMEM media, and the mixtures were added to each well at a volume equal to half the volume of growth media. Before any downstream treatments were initiated, cells were incubated with the transfection mixtures at 37 °C, 5% CO_2_ for at least 24 h to allow sufficient expression of the constructs. Polyinosinic:polycytidylic acid (poly(I:C); Tocris/BioTechne, Minneapolis, MN, USA) was also transfected with Lipofectamine 2000 in a similar manner. However, poly(I:C) was transfected at a concentration of 1 μg/mL, with 1.5 μL/mL Lipofectamine 2000. Furthermore, in these assays, transfection media of cells treated with poly(I:C) was exchanged with DMEM containing 2% FBS and pen/strep 6 h post-poly(I:C)-transfection. Morpholino (MO) oligos were transfected using Endo-Porter peptide reagent (Gene-Tools, Philomath, IN, USA). MOs were first added at a concentration of 6 μM per well and Endo-Porter was then added at 4 μM per well (see Table S1 for MO sequences). The cells then incubated at 37 °C, 5% CO_2_ for at least 16 h before any subsequent treatments.

### 2.4. Luciferase Reporter Assays

Firefly luciferase assays were performed following the manufacturer’s protocol (Gold Biotechnology, St. Louis, MO, USA). Briefly, cells transfected with luciferase reporter construct via Lipofectamine 2000 were harvested with 1× firefly luciferase lysis buffer, rocked at room temperature for at least 15 min, and lysates were then centrifuged to remove cell debris. The samples were then immediately assayed or stored at −80 °C until measurements could be obtained. When preparing the assays, firefly luciferase working solution was prepared by adding D-luciferin (10 mg/mL) to luciferase assay buffer at a ratio of 1:50. Within 1 h of preparing firefly luciferase working solution, luminescence of the samples was measured in a BioTek Gen5 instrument, and samples were measured individually. First, 20 μL of lysate was added to 100 μL of firefly luciferase working solution. Luminescence was then read with an integration time ~10 s, and the next sample was then prepared and read. Luciferase activity was measured in a white-walled 96-well plate, and to avoid cross-contamination of light during readings, samples were added to every other well of the plate. 

### 2.5. RNA Extractions and Reverse Transcription

Cells were lysed with TRIzol (Thermo Fisher Scientific) and RNA was extracted from the reagent according to the manufacturer’s protocol. To separate RNA into the aqueous layer, lysates were first treated with 0.2 mL chloroform per 1 mL TRIzol. Following vortex and incubation at room temperature for 10 min, the lysates were spun at 12,000× *g* and 4 °C for 15 min. Aqueous layers containing RNA were then transferred to new tubes, and to precipitate the RNA, 2.5 μL of 4 mg/mL glycogen and 0.5 mL isopropanol per 1 mL of TRIzol used were added. After vortex and incubation at room temperature for 10 min, RNA samples were then spun at 12,000× *g* and 4 °C for 10 min. Supernatant was then removed, and the RNA pellets were washed with 1 mL of 75% ethanol per 1 mL of TRIzol used. Washes were accomplished by brief vortex of the pellets in 75% ethanol and centrifugation at 7600× *g* and 4 °C for 5 min. 75% ethanol solution was then removed, and the pellets dried at room temperature for 5–10 min. RNA was then resuspended in nanopure H_2_O, incubated at 55–60 °C for 10–15 min., and stored at −80 °C. Extracted RNA was then reverse transcribed to cDNA with Maxima H Minus Reverse Transcriptase (Thermo Fisher Scientific). Prior to reverse transcription, RNA in each sample was diluted to ~100 ng/μL. Diluted RNA was incubated with random hexamer primers and dNTPs at 65 °C for 5 min., chilled, and reverse transcription reagents (reverse transcription buffer and Maxima H Minus Reverse transcriptase) were then added. After addition of reverse transcription reagents, samples were incubated at 25 °C for 10 min., 50 °C for 30 min., and 85 °C for 5 min. Resulting cDNA was then either immediately used or stored at −20 °C. 

### 2.6. RT-qPCR

RT-qPCR measurements were obtained using SYBR Green Master Mix and the CFX Connect Real-Time PCR Detection System (Bio-Rad, Hercules, CA, USA). First, cDNA was diluted 1:5 in nanopure H_2_O. Measurements were obtained using a 384-well plate, and each well with sample contained 5 μL SYBR Green Master Mix, 1 μL forward + reverse 10 μM primer mix, and 4 μL diluted cDNA. cDNA was then amplified and measured under the following conditions: (1) 50 °C for 2 min., (2) 95 °C for 2 min., (3) 95 °C for 15 s, (4) 60 °C for 1 min., (5) Plateread, (6) Go to (3) 39×, (7) 65 °C for 31 s, (8) 65 °C for 5 s + 0.5 °C/cycle Ramp 0.5 °C/s, (9) Plateread, (10) Go to 8) 60×. Relative normalized expression was quantified in CFX Maestro (Bio-Rad) using the ∆∆Ct method, and RNA was normalized to GAPDH (see Table S1 for qPCR primer sequences). 

### 2.7. Gel Electrophoresis

Prior to electrophoresis, cDNA of RIOK3 and its alternatively spliced isoforms were amplified via PCR InPhusion Flash Hi-Fidelity PCR Master Mix (Thermo Fisher Scientific) (see Table S1 for PCR primer sequences). Each reaction mixture consisted of 10 μL Phusion Flash PCR Master Mix, 1 μL of 10μM RIOK3 TotEx5 primer, 1 μL of 10 μM RIOK3 TotEx10 primer, 6 μL nanopure H_2_O, and 2 μL cDNA. cDNA was then amplified under the following conditions: (1) 98 °C for 10 s, (2) 98 °C for 1 s, (3) 65 °C for 5 s, (4) 72 °C for 15 s, (5) Go to (2) 29×, (6) 72 °C for 1 min., (7) 4 °C forever. RIOK3, RIOK3 X2, and RIOK3 X1/X2 hybrid cDNAs were then resolved on a 1% agarose gel at 80 V. After rocking for 10 min. in a 1× TAE bath containing ethidium bromide, images were obtained via Gel Doc XR + Molecular Imager (Bio-Rad). 

### 2.8. Statistical Analysis

Statistically significant differences in the data were determined using a two-tailed unpaired Student’s *t*-test in GraphPad Prism 8. Error bars are reported as standard error of the mean. 

## 3. Results

### 3.1. RIOK3 Has Distinct Roles in the Cellular IFN and NFκB Responses 

**Figure 2:**
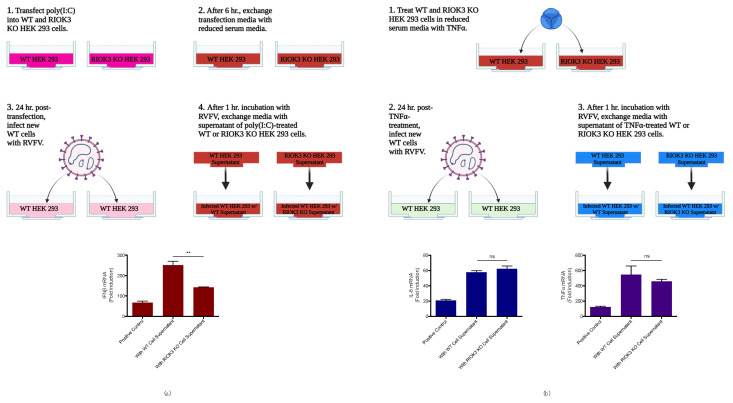
Paracrine communication from cells deficient in RIOK3 elicits diminished IFN response to infection with RVFV. (**a**) WT or RIOK3 KO cells were immune stimulated with poly(I:C) (1μg/mL), and media from these cells was transferred to identical plates of WT cells infected with RVFV (see diagram). IFNβ mRNA expression in the infected cells was measured by RT-qPCR. Cells treated with media from the WT cells elicited a stronger IFN response to infection than cells treated with media from RIOK3 KO cells. (**b**) Similarly, WT or RIOK3 KO cells were treated with TNFα at a concentration of 20 ng/mL, and media from these cells was transferred to identical plates of WT cells infected with RVFV (see diagram). Then, IL-8 and TNFα mRNA expression was measured by RT-qPCR. Cells treated with media from the WT or RIOK3 KO cells did not elicit statistically different inflammatory responses to infection, as measured by expression of IL-8 and TNFα. Fold induction in plots is relative to values of cells mock-infected and incubated with reduced serum media (negative controls). In (**a**,**b**), cells were grown in 6-well plates, seeded at a density ~0.3 × 10^6^ cells/well, and infected at a multiplicity of infection (MOI) ~2. Plots present the data as the mean value of 3 biological replicates +/− SEM. Student’s *t*-test: ** *p* < 0.01.

Although we and others have shown that RIOK3 plays an important role in the cellular response to RVFV infection, there is still not general consensus on the exact roles of RIOK3 in the IFN and inflammatory responses. Therefore, we employed a previously unused strategy that tests how RIOK3 affects the ability of cells to alert other cells to viral (or viral mimetic) threats in a paracrine fashion. During an innate immune response, cytokines are released into the surroundings to alert neighboring cells of the infection. For example, during the IFN response, IFNβ protein is released to activate the JAK/STAT pathway in autocrine and paracrine communications. During an inflammatory response, TNFα is released to the surroundings, resulting in further activation of the NFκB-mediated inflammatory pathway [38,66]. Exposure of cells to cytokines released from other immune stimulated cells brings them to an alerted state that results in a more robust immune response to an incoming pathogen. By extension, any reduction in release of cytokines by a cell subjected to immune activation could diminish the preparedness of adjacent cells against imminent infection. Here, to test whether RIOK3 impacts the secretion of cytokines leading to IFN and/or inflammatory responses in surrounding cells, we used the (cytokine-containing) media of stimulated wt or RIOK3 KO cells to treat identical plates of RVFV MP12 infected wild type cells (Figure 2). If RIOK3 plays a role in expression of secreted cytokines, media from stimulated cells lacking RIOK3 should stimulate a less robust response against subsequent infection. This was true for the IFN response, but did not affect inflammatory pathway gene transcription (Figure 2). 

WT and RIOK3 KO HEK 293 cells were first transfected with poly(I:C), which contains a mixture of short and long dsRNAs recognized by both RIG-I and MDA5 [2,67,68], to activate the IFN pathway. After 24 h of poly(I:C) treatment, media from WT or RIOK3 KO cells treated with poly(I:C) was added to RVFV-infected WT HEK 293 cells that had incubated with virus for 1 h (experimental scheme is diagramed in Figure 2a). WT cells infected with RVFV and incubated with standard (i.e., cytokine-free) reduced serum media (2% FBS with pen/strep) served as positive controls, and WT cells mock-infected and incubated with the same media served as negative controls. Relative expression of IFNβ mRNA was then measured by RT-qPCR, and a statistically significant decrease in IFNβ expression was observed in RVFV-infected cells incubated with supernatant from RIOK3 KO cells compared to RVFV-infected cells incubated with supernatant from WT cells. These results suggest an impairment of IFN (and potentially other cytokines) release from RIOK3 KO cells (Figure 2a). Further, we found that TCID50/mL values were elevated in infected cells incubated with media from poly(I:C)-treated RIOK3 KO cells. This showed that more (or more active) virus was produced in WT cells treated with culture media from the KO cells, and also indicated the decreased IFNβ transcription in these cells was not due to an impairment in RVFV replication and spread (Figure S1a). These results also suggest that the absence of RIOK3 in the IFN response could be favorable for RVFV. Collectively, these results support the hypothesis that RIOK3 does have a role in mounting the IFN response, including in the paracrine communication between cells. 

To test whether RIOK3 has a role in regulating secreted cytokines involved in the inflammatory response to TNFα, WT and RIOK3 KO HEK 293 cells were treated with TNFα protein for 24 h. This treatment results in activation of the canonical NFκB pathway and release of inflammatory cytokines into the media that can further activate the canonical NFκB pathway, including transcription of more TNFα [58,59,60,61]. Separate WT HEK 293 cells were then infected with RVFV and incubated for 24 h with supernatant from TNFα-treated WT cells or with supernatant from TNFα-treated RIOK3 KO cells (experimental scheme is diagramed in Figure 2b). Positive and negative controls were identical to those in Figure 2a. Relative normalized expression of IL-8, a target gene of NFκB in response to TNFα protein [69] (also see Figure S2), and TNFα mRNA were quantified. No statistically significant differences in expression of these inflammatory cytokines were observed between RVFV-infected cells incubated with supernatant from TNFα-treated WT cells vs. infected cells incubated with supernatant from TNFα-treated RIOK3 KO cells (Figure 2b). Thus, absence of RIOK3 does not seem to affect inflammatory cytokine release in response to TNFα. Intriguingly, TCID50 assays showed increased infectious dose of RVFV MP12 in infected cells incubated with media from TNFα-treated RIOK3 KO cells, suggesting absence of RIOK3 in the TNFα-triggered inflammatory response could be favorable for RVFV (Figure S1b). Overall, these results suggest that the role RIOK3 might play in intercellular/paracrine activation of the inflammatory pathway is subtle.

### 3.2. RIOK3 Has a Role in Negatively Regulating the NFκB-Mediated Inflammatory Response during RVFV MP12 Infection

**Figure 3:**
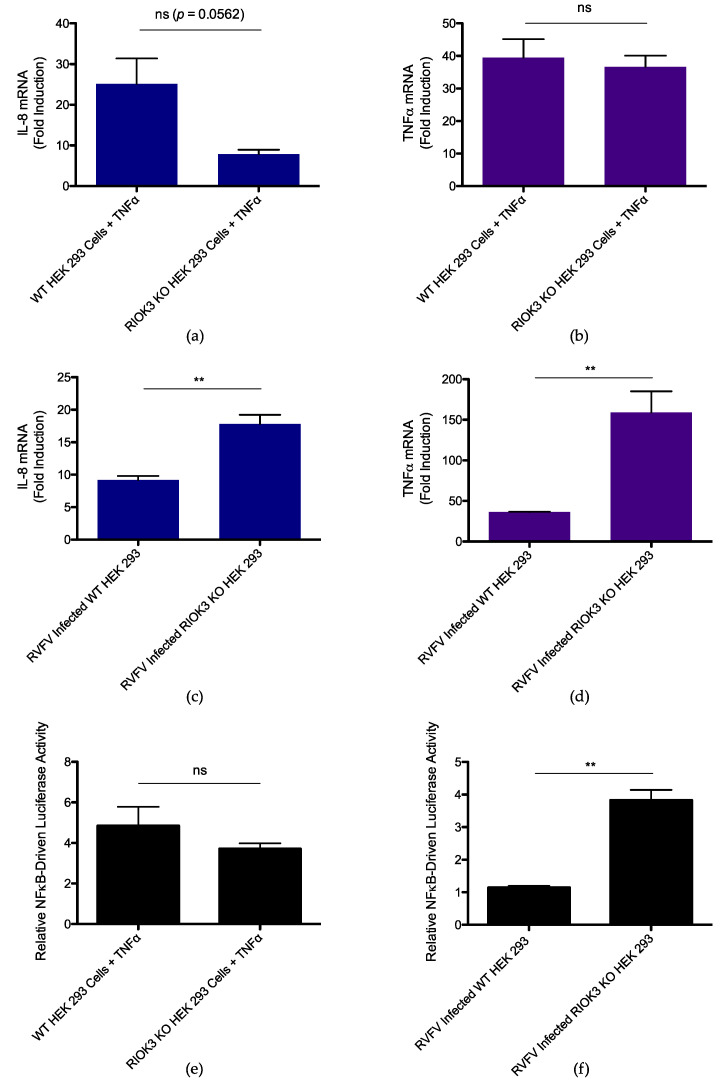
Effect of RIOK3 KO on inflammatory cytokine transcription during TNFα treatment or RVFV infection. (**a**) IL-8 mRNA expression in WT vs. RIOK3 KO cells treated with 20 ng/mL TNFα for 4 h. (**b**) TNFα mRNA expression in WT vs. RIOK3 KO cells treated with 20 ng/mL TNFα for 4 h. (**c**) IL-8 mRNA expression in WT vs. RIOK3 KO cells infected with RVFV MP12 at an MOI ~1. (**d**) TNFα mRNA expression in WT vs. RIOK3 KO cells infected with RVFV MP12 at an MOI ~1. (**e**) Luciferase assay measuring NFκB promoter activity in WT vs. RIOK3 KO HEK 293 cells treated with 20 ng/mL TNFα for 4 h. Fold induction is relative to negative control values from respective cell types (WT or RIOK3 KO) that were not treated with TNFα. (**f**) Luciferase assay measuring NFκB promoter activity in WT vs. RIOK3 KO HEK 293 cells infected with RVFV at an MOI ~2. Fold induction is relative to negative control values from respective cell types (WT or RIOK3 KO) that were mock-infected. In (**a**,**b**), cells were grown in 12-well plates and seeded at a density ~0.1 × 10^6^ cells/well, and fold induction is relative to expression values of respective cell types that were not treated with TNFα. In (**c**,**d**), fold induction is relative to expression values of respective cell types that were mock-infected. Plots present the data as the mean value of 3 biological replicates +/− SEM. Student’s *t*-test: ** *p* < 0.01.

Previous studies have suggested both positive and negative roles for RIOK3 in the inflammatory response [63]. To more directly explore how RIOK3 KO affects the NFκB-mediated inflammatory response, we investigated how TNFα treatment and RVFV infection affected WT and RIOK3 KO cells. First, WT and RIOK3 KO HEK 293 cells were treated with TNFα for 4 h, and relative expression of IL-8 and TNFα were then measured by RT-qPCR. Fold inductions were measured relative to respective cell types not treated with TNFα. Similar to what was observed in Figure 2b, no statistically significant differences in relative expression of IL-8 or TNFα were observed between WT and RIOK3 KO cells (Figure 3a,b). This suggests absence of RIOK3 does not affect net transcription of inflammatory cytokines when the canonical NFκB pathway is activated with TNFα. However, differences in the inflammatory response in WT vs. RIOK3 KO cells triggered by viral infection were observed (see below). 

To test how RIOK3 absence affects expression of inflammatory cytokines in response to RVFV, WT and RIOK3 KO HEK 293 cells were infected with RVFV MP12. 24 h post-infection (h.p.i.), cells were harvested, and relative expression of IL-8 and TNFα were measured. Fold induction was measured relative to respective cell types that were mock-infected. Statistically significant increases in relative expression of these cytokines were observed in RIOK3 KO cells, suggesting RIOK3 negatively regulates the inflammatory response resulting in transcription of these cytokines during RVFV infection (Figure 3c,d). 

To further investigate effects of knocking out RIOK3 on inflammatory cytokine expression stimulated by TNFα treatment and RVFV MP12 infection, we employed a luciferase reporter construct (p NFκB-Luc) that is specifically driven by activated NFκB. WT and RIOK3 KO HEK 293 cells were transfected with the reporter construct to specifically monitor NFκB targeted promoter activation. First, the effect of RIOK3 absence on NFκB target promoter activation during TNFα treatment was assessed. 24 h after transfecting WT and RIOK3 KO cells with the reporter construct, cells were treated with TNFα for 4 h. Consistent with Figure 2b and Figure 3a,b, no statistically significant difference in promoter activity was observed between WT and RIOK3 KO cells (Figure 3e). Furthermore, the same NFκB reporter construct was transfected into WT and RIOK3 KO cells, and 24 h post-transfection, the cells were infected with RVFV MP12. 24 h.p.i., the cells were harvested, and NFκB target promoter activity was measured. A statistically significant increase in promoter activity was observed in RVFV-infected RIOK3 KO cells compared to RVFV-infected WT cells, suggesting endogenous RIOK3 mitigates activation of the inflammatory response during viral infection. Collectively, these results demonstrate that RIOK3 negatively regulates the NFκB-mediated inflammatory response during RVFV infection, while its role in mediating the TNFα-induced inflammatory response is more nuanced (see below). 

### 3.3. Alternative Splicing of RIOK3 during RVFV Infection Correlates with Increased Inflammatory Response and Decreased IFN Response

**Figure 4:**
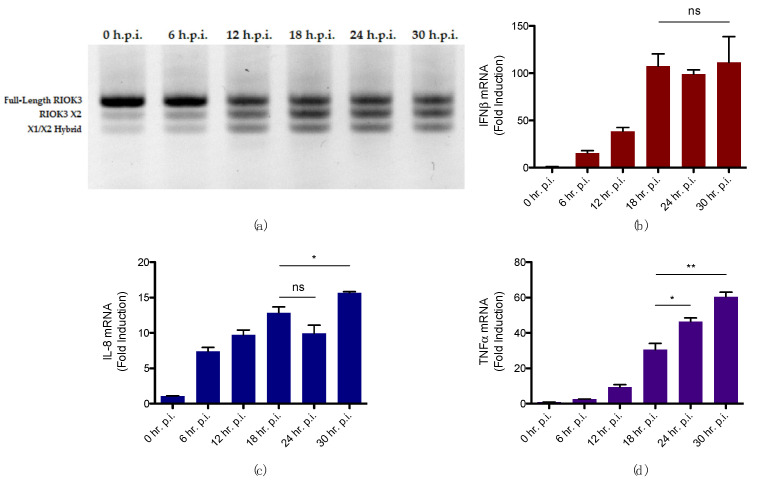
Accumulation of alternatively spliced RIOK3 mRNA isoforms during RVFV MP12 infection correlates with a stalled IFN response but rising inflammatory response. (**a**) Alternative splicing of RIOK3 in RVFV MP12-infected HEK 293cells from 0 to 30 h.p.i. as detected by RT-PCR (**b**) IFNβ mRNA expression in RVFV MP12-infected cells from 0 to 30 h.p.i. (**c**) IL-8 mRNA expression in RVFV MP12-infected cells from 0 to 30 h.p.i. (**d**) TNFα mRNA expression in RVFV MP12-infected cells from 0 to 30 h.p.i. In plots (**b**–**d**), fold induction is relative to values of cells at 0 h.p.i. Cells were grown in 12-well plates, seeded at a density ~0.1 × 10^6^ cells/well, and infected at an MOI ~2. Plots in (**b**–**d**) present the data as the mean value of 3 biological replicates +/− SEM. Student’s *t*-test: * *p* < 0.05, ** *p* < 0.01.

Although alternative splicing of RIOK3 during RVFV infection has been investigated previously in our laboratory with regard to its potential effect on the IFN response, the effect of this event on the inflammatory response during RVFV infection has not. We previously observed that, beginning early in RVFV infection, RIOK3 mRNA is alternatively spliced, primarily to the X2 isoform, which introduces a premature stop codon in exon 9, corresponding to amino acid 316 the beginning of the kinase domain. Moreover, we found alternative splicing of RIOK3 to RIOK3 X2 resulted in a diminished IFN response [24,25,26]. To understand more fully how this alternative splicing event correlates with the cellular innate immune response during RVFV infection, WT HEK 293 cells were infected with RVFV MP12 and harvested at 6, 12, 18, 24, and 30 h.p.i. Relative normalized expression of IFNβ, IL-8, and TNFα throughout infection were measured in parallel with assessment of alternative splicing at each timepoint. Alternative splicing was assessed using gel electrophoresis, which allows visualization of amplified cDNA of RIOK3 and its alternatively spliced isoforms. Up to 3 bands are typically observed in the gel: full-length (i.e., constitutively spliced) RIOK3, RIOK3 X2, and a RIOK3 X1/X2 hybrid. Full-length RIOK3 is the largest amplicon and thereby migrates through the gel the slowest. The RIOK3 X2 cDNA amplicon migrates faster than full-length RIOK3, and it is represented in the middle band in the gel. RIOK3 X1/X2 hybrid amplicon migrates the fastest and is observed at the bottom. Throughout RVFV infection, alternative splicing begins early in infection, and the X2 isoform appears to reach a peak concentration by 18 h.p.i. (Figure 4a). By this time, expression of IFNβ ceases to rise (Figure 4b). However, expression of inflammatory cytokines continues to increase (Figure 4c,d).

These results support the hypothesis that full-length RIOK3 protein activates the IFN response and inhibits the inflammatory response in RVFV infections. Furthermore, the appearance of truncated isoform X2, is associated with inhibition the IFN response and activation of the inflammatory response. It is likely that both the diminution of full length RIOK3 as a result of the shift toward the X2 isoform, and the appearance of X2 per se contribute to these effects on the IFN and inflammatory pathways, as evidenced by the X2 expression experiments below.

### 3.4. RIOK3 X2 Expression Augments TNFα-Induced Inflammatory Pathway Activation 

**Figure 5:**
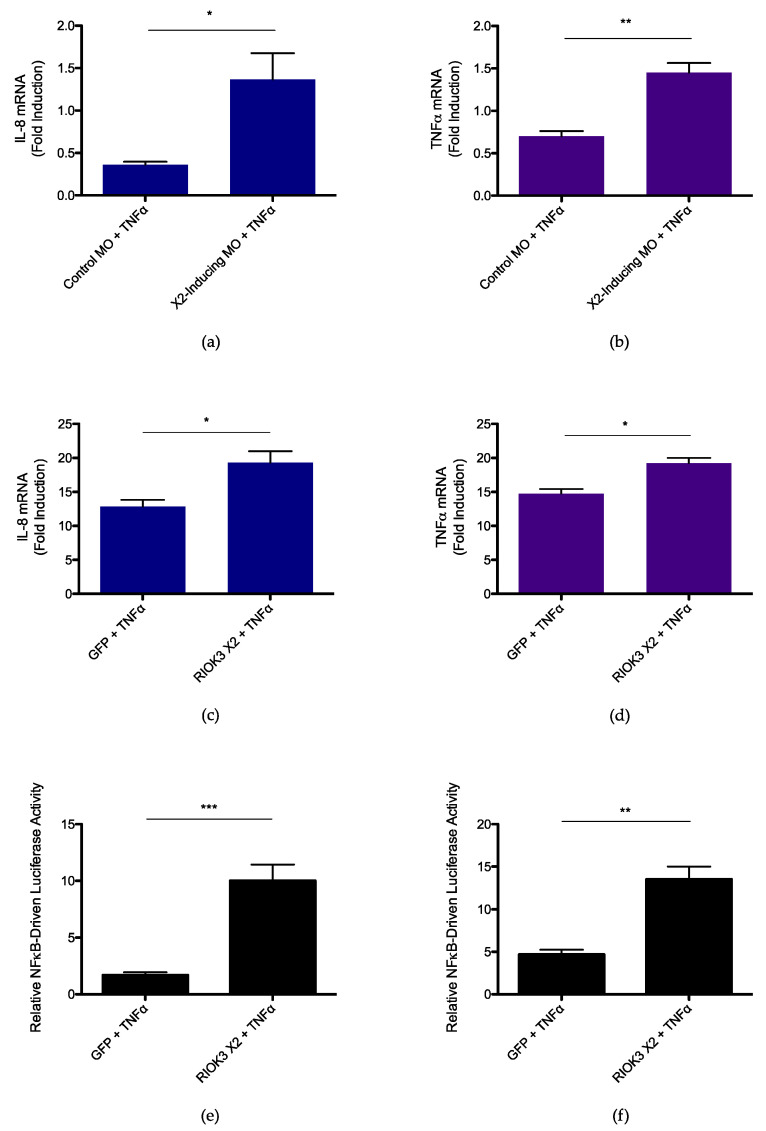
RIOK3 X2 expression triggers inflammatory pathway activation. (**a**) IL-8 mRNA expression in X2-inducing MO- vs. standard control MO-treated WT HEK 293 cells (**b**) TNFα mRNA expression in X2-inducing MO- vs. standard control MO-treated WT HEK 293 cells (**c**) IL-8 mRNA expression in GFP- vs. RIOK3 X2-transfected WT HEK 293 cells (**d**) TNFα mRNA expression in GFP- vs. RIOK3 X2-transfected WT HEK 293 cells (**e**) Luciferase assay measuring NFκB promoter activity in GFP- vs. RIOK3 X2-transfected WT HEK 293 cells (**f**) Luciferase assay measuring NFκB promoter activity in GFP- vs. RIOK3 X2-transfected RIOK3 KO HEK 293 cells. In plots (**a**,**b**), fold induction is relative to values of cells transfected with standard control MO and that were not treated with TNFα. Cells were grown in 12-well plates, seeded at a density ~0.2 × 10^6^ cells/well, and treated with 80 ng/mL TNFα for 4 h. In (**c**,**d**), fold induction is relative to values of cells transfected with GFP and that were not treated with TNFα. In (**c**–**f**), cells were grown in 12-well plates, seeded at a density ~0.1 × 10^6^ cells/well, and treated with 20 ng/mL TNFα. In (**c**–**e**), cells were treated with TNFα for 4 h, and in (**f**), cells were treated with TNFα for 24 h. Plots in (**a**–**f**) present the data as the mean value of 3 biological replicates +/− SEM. Student’s *t*-test: * *p* < 0.05, ** *p* < 0.01, *** *p* < 0.001.

To further understand how the spliced isoform RIOK3 X2 impacts the NFκB-mediated inflammatory response, we examined the effect of X2 protein expression on the inflammatory response triggered by TNFα. WT HEK 293 cells were first treated with a morpholino oligonucleotide (MO; GeneTools LLC) that, when introduced into cells, occludes the canonical splice donor site within exon 8 of RIOK3 pre-mRNA and results in abundant X2 alternative splicing [24]. 24 h after transfection of the X2-inducing MO or standard control MO, cells were treated with TNFα for 4 h. After harvest, relative normalized expression of inflammatory cytokines IL-8 and TNFα was measured by RT-qPCR. The induction of these cytokines in cells treated with TNFα was normalized to expression in cells transfected with the control MO that were not treated with TNFα. Significant increases in expression of IL-8 and TNFα were observed in cells treated with X2-inducing MO compared to cells treated with a standard control MO (Figure 5a,b). Additionally, splicing was assessed in the same lysates to confirm the X2-inducing MO was effective at the time of lysis and that alternative splicing of RIOK3 did not occur to the same degree in cells transfected with standard control MO (Figure S3). Moreover, to confirm the upregulation in inflammatory cytokine expression was specifically due to the MO targeting RIOK3 pre-mRNA, RIOK3 KO HEK 293 cells were also treated with X2-inducing MO or standard control MO and treated with TNFα for 4 h. If the increased expression of inflammatory cytokines in WT cells transfected with X2-inducing MO is due to alternative splicing of RIOK3, this result would not be expected in cells lacking an intact RIOK3 gene, and in fact, cytokine expression was similar between TNFα-treated RIOK3 KO cells transfected with X2-inducing MO vs. standard control MO (Figure S4).

Since alternative splicing of RIOK3 decreases expression of full-length RIOK3 transcripts at the expense of X2 mRNA, the increased inflammatory response observed in cells treated with X2-inducing MO could be either due to decreased RIOK3 protein levels and/or due to a direct effect of translated RIOK3 X2 protein itself. Prior work in our laboratory has shown that RIOK3 X2 mRNA is a canonical target of nonsense-mediated decay, and the truncated protein arising from translation of RIOK3 X2 appears to have a short half-life, suggesting expression of RIOK3 X2 is stringently regulated by the cell [24] as would be expected for an inflammatory protein [70]. To test whether RIOK3 X2 protein could directly enhance the inflammatory response to TNFα, the RIOK3 X2 expression construct was transfected into WT HEK 293 cells, and the cells were treated with TNFα for 4 h. Cells transfected with an enhanced GFP (eGFP) expression construct and treated with TNFα served as positive controls, and cells transfected with eGFP but were untreated served as negative controls. Expression of IL-8 and TNFα mRNA was measured via RT-qPCR, and significant increases in expression were observed in TNFα-treated cells transfected with RIOK3 X2 (Figure 5c,d). Moreover, RIOK3 X2 was also co-transfected with the NFκB luciferase reporter construct into WT cells, and 24 h after transfection, the cells were treated with TNFα for 4 h. Similar positive and negative controls as those in Figure 5c,d were used, and significant increases in NFκB promoter activity was observed in cells transfected with RIOK3 X2 (Figure 5e). Furthermore, RIOK3 X2 was also co-transfected with the NFκB luciferase reporter construct into RIOK3 KO cells, and 24 h after transfection, the cells were treated with TNFα for 24 h. Again, significant increases in NFκB promoter activity were observed in cells transfected with RIOK3 X2 (Figure 5f). 

Collectively, these results suggest alternative splicing of RIOK3 enhances the NFκB-mediated inflammatory response, ostensibly due to a direct effect of translated RIOK3 X2 protein on this response.

## 4. Discussion

RIOK3 plays important yet enigmatic roles in the innate immune response to RNA virus infection, as evidenced by disparate results from different studies. In this work, we have begun to shed light on the roots of these discrepancies by demonstrating that RIOK3 has different effects on separate branches of the innate immune response. As we and others have shown previously, RIOK3 plays a key role in eliciting an IFN response and here we also show it likely negatively regulates the inflammatory response. Surprisingly, alternative splicing of RIOK3 mRNA to express the RIOK3 X2 isoform reverses these roles, resulting in a mitigated IFN response and an elevated inflammatory NFκB response. This alternative splicing event appears to be favorable for RVFV MP12 infection in cell culture, and it is conceivable that enhancing or co-opting this alternative splicing is part of RVFV’s strategy to manipulate host innate immunity. However, whether these results are restricted to the attenuated RVFV strain used in these experiments opposed to WT strain still needs to be addressed. 

The diminished IFN response and elevated inflammatory response observed when RIOK3 is alternatively spliced to RIOK3 X2 is likely due to both decreased full-length RIOK3 and a direct effect of RIOK3 X2 peptide based on our RIOK3 KO and X2 direct expression experiments. Although the mechanism for how expression of the truncated form of RIOK3 exerts an effect in the IFN and NFκB pathways is not yet fully understood, this event does appear to be consequential for the cell during viral infection, and could contribute to pathology. Indeed, several RNA viruses, including SARS CoV-2, apparently piggyback on the expression of inflammatory cytokines as part of their replication strategies (see, e.g., [71,72]). By demonstrating that RIOK3 has different effects in separate innate immune pathways, qualitative or quantitative changes to its expression during viral infection could very well lead to dysregulated innate immune responses that are harmful to the host and/or are designed to enhance viral success.

Other innate immune proteins having distinct roles in the IFN and inflammatory pathways have also been documented. For example, similar to what we have found with RIOK3, TBK1 is responsible for activating the IFN response, but it also has been shown to negatively regulate NFκB pathways [73]. Moreover, alternatively spliced isoforms of immune modulators that have distinct effects from their counter-isoform have also been described. For example, IKKγ is known to facilitate activation of the IFN pathway, but its alternatively spliced isoform IKKγ∆ has been shown to be incapable of this [74].

Since we have shown that RIOK3′s alternatively spliced isoform, RIOK3 X2, has distinct effects on the immune response compared to RIOK3, this isoform needs to be taken into consideration when deciphering the roles of RIOK3 in the innate immune response to RNA viruses. In particular, some of the current discrepancies in the literature of what effect RIOK3 has on innate immunity could in part be explained by the interplay of RIOK3 and RIOK3 X2. For example, RIOK3 X2′s effect on the NFκB pathway could be a reason why Fenner et al. [63] found RIOK3 both activates and inhibits this pathway. However, even though the results obtained here suggest that RIOK3 exhibits a negative effect on this response, full-length RIOK3 might still enhance this inflammatory pathway at certain times of the response. Cataloging interaction partners of RIOK3 during innate immune activation will be important for addressing this and understanding the various modes of function RIOK3 has in innate immune pathways. 

**Figure 6:**
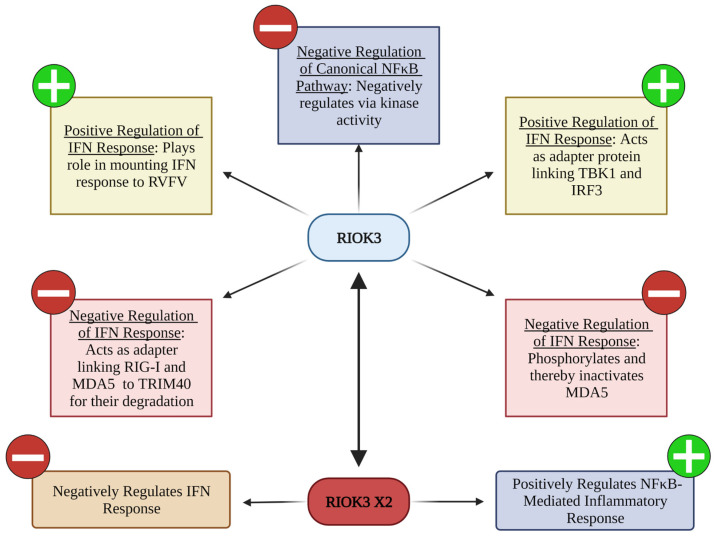
Current knowledge of RIOK3′s modulation of innate immunity to RNA virus infection.

A model encompassing what is currently known about RIOK3′s involvement in the innate immune responses to RNA virus infection is shown in Figure 6. Considering RIOK3 plays important roles in the innate immune response to RNA viruses, this atypical kinase certainly warrants continued investigation to further untangle the intricacies and nuances of these responses. The results presented here shed light on the regulatory potential of RIOK3 and portend new discoveries of its roles during RVFV and other RNA virus infections. 

## Data Availability

Not applicable.

## Supplementary Materials

The following supporting information can be downloaded at: https://www.mdpi.com/article/10.3390/v14092064/s1, Table S1: Sequences of oligonucleotides used; Figure S1. Infectious dose of RVFV MP12 in infected WT HEK 293 cells incubated with supernatant from poly(I:C)- or TNFα-treated cells; Figure S2: Relative normalized expression of (a) IL-8 and (b) TNFα mRNA in response to TNFα protein; Figure S3: Alternative splicing of RIOK3 in standard control MO- vs. X2-inducing MO-treated cells; Figure S4: Relative normalized expression of (a) IL-8 and (b) TNFα mRNA in RIOK3 KO cells treated with standard control MO vs. X2-inducing MO.
