# Supplementary material for: RIOK3 and Its Alternatively Spliced Isoform Have Disparate Roles in the Innate Immune Response to Rift Valley Fever Virus (MP12) Infection
Source: Viruses. 2022 Sep 17;14(9):2064. doi: 10.3390/v14092064 (PMC9502082; doi:10.3390/v14092064)
Supplement: Supplementary file 1 [file viruses-14-02064-s001.zip › viruses-1924654-supplementary.pdf]

Table S1. Sequences of oligonucleotides used. TNFα qPCR primers used were predesigned by Integrated DNA Technologies (IDT) and spanned exons 1b-4a.

qPCR Primer	qPCR Primer Sequence
F_IFNβ	5′-AAACTCATGAGCAGTCTGCA-3′
R_ IFNβ	5′-AGGAGATCTTCAGTTTCGGAGG-3′
F_IL-8	5′-GGCAGCCTTCCTGATTTCTG-3′
R_IL-8	5′-CTTGGCAAAACTGCACCTTCA-3′
F_GAPDH	5′-GTCTCCTCTGACTTCAACAGCG-3′
R_GAPDH	5′-ACCACCCTGTTGCTGTAGCCAA-3′
**PCR Primer**	**PCR Primer Sequence**
RIOK3 Exon 5	5′-CCGGTTCCCACTCCTAAAAAGGGC-3′
RIOK3 Exon 10	5′-CCAGCATGCCACAGCATGTTATACTCA-3′
**Morpholino Oligo**	**Morpholino Oligo Sequence**
RIOK3 X2-Inducing	5′-ATTTTGCTCATTTACCTCCCTCCAT-3′
Standard Control	5′-CCTCTTACCTCAGTTACAATTTATA-3′

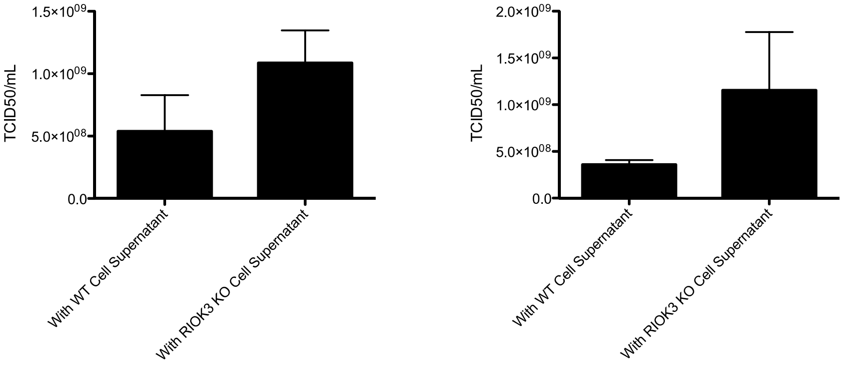

(a) (b)

Figure S1. Infectious dose of RVFV MP12 in infected WT HEK 293 cells incubated with supernatant from poly(I:C)- or TNFα-treated cells. (**a**) TCID50/mL values of infected cells incubated with supernatant from poly(I:C)-treated WT or RIOK3 KO HEK 293 cells. (**b**) TCID50/mL values of infected cells incubated with supernatant from TNFα -treated WT or RIOK3 KO HEK 293 cells. Plots present the data as the mean value of 3 biological replicates +/- SEM.

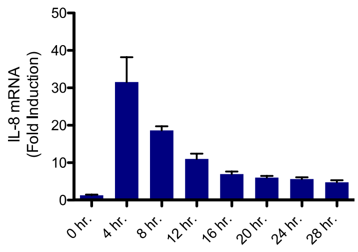

(a)

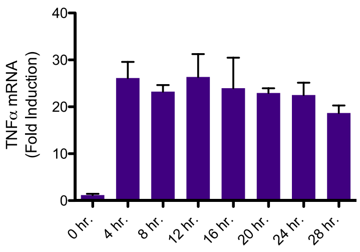

(b)

Figure S2. Relative normalized expression of (**a**) IL-8 and (**b**) TNFα mRNA in response to TNFα protein. WT HEK 293 cells were treated with 20 ng/mL TNFα and lysed at 4 hr. intervals from 0 to 28 hr. post-treatment. Fold induction is relative to WT cells that were not treated with TNFα. Cells were grown in 12-well plates and seeded at a density ~0.1 x 10^6 cells/well. Plots present the data as the mean value of 3 biological replicates +/- SEM.

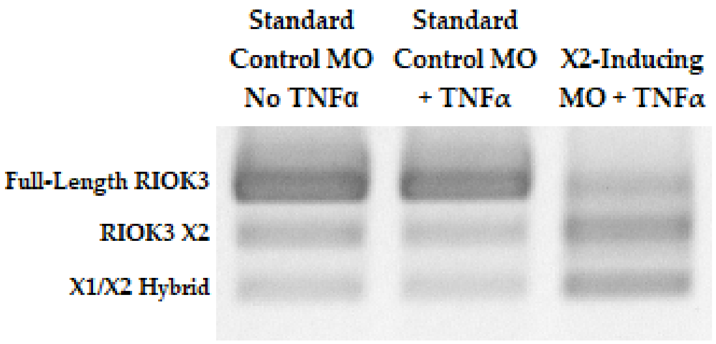

Figure S3. Alternative splicing of RIOK3 in standard control MO- vs. X2-inducing MO-treated cells. For information on seeding density, see **Figure 6** caption in the main text.

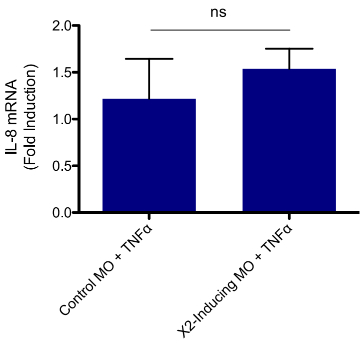

(a)

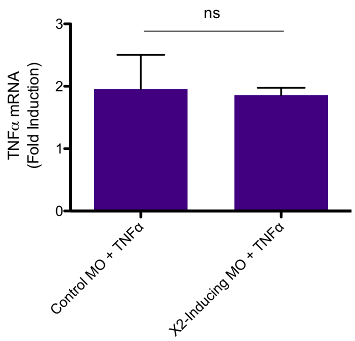

(b)

Figure S4. Relative normalized expression of (**a**) IL-8 and (**b**) TNFα mRNA in RIOK3 KO cells treated with standard control MO vs. X2-inducing MO. Fold induction is relative to values of cells transfected with standard control MO and that were not treated with TNFα. Cells were grown in 12-well plates, seeded at a density ~0.2 x 10^6 cells/well, and treated with 80 ng/mL TNFα.
